# Development of encoded Broccoli RNA aptamers for live cell imaging of alphavirus genomic and subgenomic RNAs

**DOI:** 10.1038/s41598-020-61573-3

**Published:** 2020-03-23

**Authors:** Voraphoj Nilaratanakul, Debra A. Hauer, Diane E. Griffin

**Affiliations:** 1 W. Harry Feinstone Department of Molecular Microbiology and Immunology, Johns Hopkins Bloomberg School of Public Health, Baltimore, MD 21205 USA; 2 Cellular and Molecular Medicine Graduate Program, Johns Hopkins University School of Medicine, Baltimore, MD 21205 USA; 3 Present Address: Division of Infectious Diseases, Department of Medicine, Faculty of Medicine, Chulalongkorn University and King Chulalongkorn Memorial Hospital, Thai Red Cross Society, Bangkok, Thailand

**Keywords:** Microbiology, Alphaviruses

## Abstract

Sindbis virus (SINV) can infect neurons and cause encephalomyelitis in mice. Nonstructural proteins are translated from genomic RNA and structural proteins from subgenomic RNA. While visualization of viral proteins in living cells is well developed, imaging of viral RNAs has been challenging. RNA aptamers that bind and activate conditional fluorophores provide a tool for RNA visualization. We incorporated cassettes of two F30-scaffolded dimers of the Broccoli aptamer into a SINV cDNA clone using sites in nsP3 (genomic RNA), the 3′UTR (genomic and subgenomic RNAs) and after a second subgenomic promoter resulting in 4–28 Broccoli copies. After addition of the cell-permeable 3,5-difluoro-4-hydroxybenzylidene imidazolinone (DFHBI-1T) conditional fluorophore and laser excitation, infected cells emitted green fluorescence that correlated with Broccoli copy numbers. All recombinant viruses replicated well in BHK and undifferentiated neural cells but viruses with 14 or more Broccoli copies were attenuated in differentiated neurons and mice. The signal survived fixation and allowed visualization of viral RNAs in differentiated neurons and mouse brain, as well as BHK cells. Subgenomic RNA was diffusely distributed in the cytoplasm with genomic RNA also in perinuclear vesicle-like structures near envelope glycoproteins or mitochondria. Broccoli aptamer-tagging provides a valuable tool for live cell imaging of viral RNA.

## Introduction

Sindbis virus (SINV), an alphavirus in the *Togaviridae* family, is an enveloped plus-strand RNA virus that can cause seasonal outbreaks of rash and arthritis in humans and encephalomyelitis in experimentally infected mice^1–3^. On entry, nonstructural replicase proteins (nsPs) are translated from the capped and polyadenylated 11.7 kb genomic RNA as two polyproteins (P123 and P1234) to initiate infection. P123 and the nsP4 RNA-dependent RNA polymerase induce spherules at the plasma membrane and replicate the full-length minus-strand RNA in early replication complexes as a template for synthesis of plus-strand RNAs^4^. P123 is subsequently processed by the nsP2 protease to nsP1 and P23 and then to individual nsPs leading to cessation of minus-strand synthesis. With the switch to stable plus-strand replication complexes genomic and subgenomic (sg) RNAs are synthesized^5,6^. sgRNA is translated to produce the structural proteins E1, E2 and capsid required for assembly of virions containing genomic RNA^5,7–12^.

To aid in studies of the regulation of viral RNA synthesis, we previously applied RNA aptamer technology to develop tagged fluorescent SINV RNA. RNA aptamers can bind and activate a conditional fluorophore that emits fluorescence in response to excitation with light at specific wavelengths^13–16^. The RNA aptamer Spinach2 within a tRNA scaffold was incorporated into a cDNA clone of the TE strain of SINV to produce recombinant viruses that expressed viral RNA tagged with the aptamer^17^. Spinach2 tagging allowed visualization of SINV RNA in live cells as green fluorescence after addition of the non-toxic cell-permeable conditional fluorophore DFHBI-1T ((Z)-4-(3,5-difluoro-4-hydroxybenzylidene)-2-methyl-1-(2,2,2-trifluoroethyl)-1H-imidazol-5(4H)-one) and excitation at 488 nm^17^. However, this system had several limitations. First, stop codons are present in all frames of tRNA-Spinach2 precluding insertion into viral protein coding regions and limiting the RNA tagging sites. Therefore, for SINV the Spinach2 aptamers were only inserted into the 3′UTR and/or after an engineered second subgenomic promoter and did not allow differentiation between viral genomic and subgenomic RNAs. Second, the signal was too dim for visualization in a conventional epifluorescence microscope and even with laser-scanning confocal microscopy, the signal remained insufficiently bright for visualization in cells with restricted replication and background autofluorescence, such as differentiated neurons.

**Figure 1:**
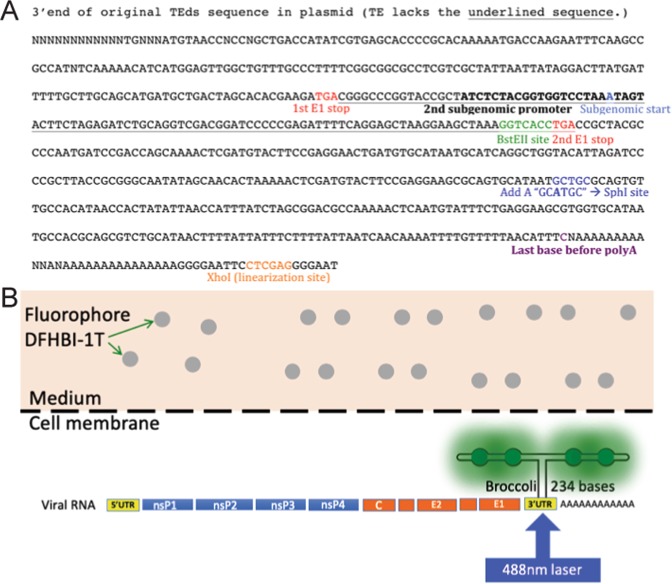
Sites for insertion and production of the SINV-Broccoli fluorescent signal. (**A**) Sequence of the 3′end of SINV TE or TEds showing sites of F30-2xdBroccoli (234 bases) insertion at the *Spe*I site in nsP3 (not shown) and/or at the *Bst*EII site (green characters) downstream of the second subgenomic promoter (bold characters) and/or at the *Sph*I site in the 3′UTR created by site-directed mutagenesis (blue characters). (**B**) Example of a constructed viral RNA genome with F30-2xdBroccoli inserted in the 3′UTR inside infected cells. Four copies of Broccoli RNA aptamers bind the cell-permeable fluorophore in the medium and emit green fluorescence upon blue laser excitation.

Recently, Broccoli, a smaller and brighter aptamer in a different scaffold than Spinach2, has been developed^18,19^. Unlike the Spinach2 tRNA scaffold, the new more stable F30 Broccoli scaffold is not subjected to intracellular cleavage and accommodates two sets of dimeric Broccoli (dBroccoli)^20,21^. The F30-2xdBroccoli (4 copies of Broccoli in total) is compact (234 bases) and contains no in-frame stop codons. After binding to the Broccoli RNA aptamer and laser excitation, the fluorophore DFHBI-1T emits green fluorescence similar to green fluorescent protein (excitation/emission = 472/507)^15,18,21^ (Fig. 1). To determine the applicability of this system to analysis of viral RNA dynamics, we constructed and characterized SINVs with different F30-2xdBroccoli numbers and insertion sites. These recombinant viruses were then used for analysis of RNA synthesis and distribution after infection of neural and non-neural cells.

## Results

### Construction of SINV-Broccoli

**Figure 2:**
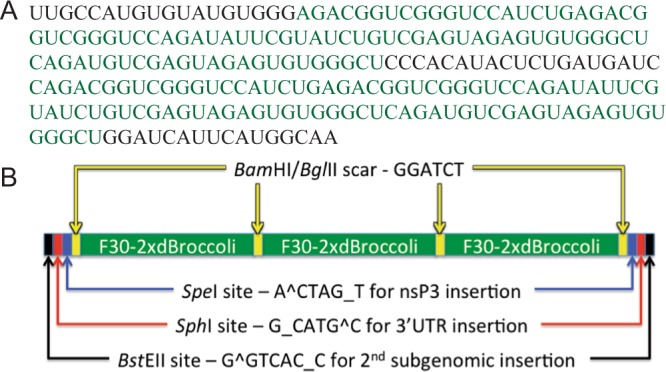
F30-2xdBroccoli and construction of tandem repeats. (**A**) Sequence of F30-2xdBroccoli with 2 copies of Broccoli dimers (bold green characters) in the F30 scaffold (black characters). (**B**) Tandem repeats consist of 3 sets of F30-2xdBroccoli (12 Broccoli in total), made from self-ligation between the complementary 5′ (*Bam*HI – G^GATC_C) and 3′ end (*Bgl*II – A^GATC_T). The ligation forms a scar (GGATCT), which can no longer be cut by either *Bam*HI or *Bgl*II. The tandem repeats can be inserted into 3 restriction sites of TEds plasmid in the specific order – nsP3, 3′UTR, and downstream of 2^nd^ subgenomic promoter.

**Figure 3:**
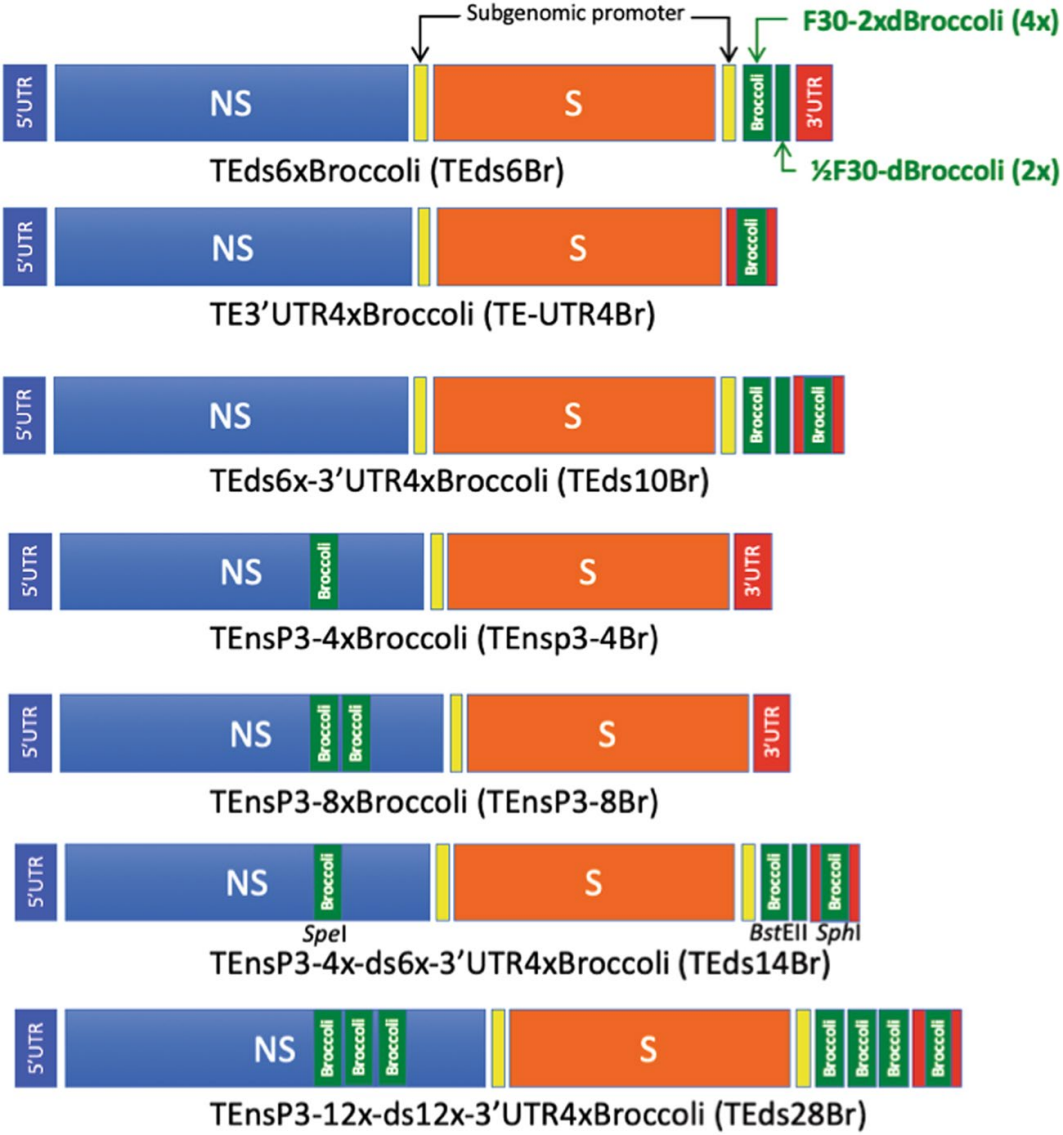
Construction of multiple SINV-Broccoli recombinant viruses. 1–3 repeats of F30-2xdBroccoli were inserted into 3 restriction sites: *Spe*I in nsP3, *Bst*EII downstream of the 2^nd^ subgenomic promoter, and *Sph*I in the 3′UTR.

To construct TE strain SINV cDNAs with Broccoli insertions, plasmids with either full-length TE^22^ or TE engineered with a second subgenomic promoter (TEds)^23^ were used. F30-2xdBroccoli, a double dimeric Broccoli (4 copies in the F30 scaffold) from the pET28c plasmid (Addgene 66843) was inserted into at least 1 of 3 restriction sites: *Sph*I engineered into the 3′UTR^17^, *Bst*EII just downstream of the second subgenomic promoter^23^, and *Spe*I in nsP3^24^ (Figs. 1A and 2). The constructed plasmids were TEds6xBroccoli (TEds6Br; the ligation resulted in 1½ sets of F30-2xdBroccoli for an unclear reason), TE3′UTR4xBroccoli (TE-UTR4Br), TEds6x-3′UTR4xBroccoli (TEds10Br), TEnsP3-4xBroccoli (TEnsp3-4Br), TEnsP3-8xBroccoli (TEnsP3-8Br), and TEnsP3-4x-ds6x-3′UTR4xBroccoli (TEds14Br) (Fig. 3). In addition, a tandem repeat was constructed as previously described^25^. Briefly, *Bam*HI and *Bgl*II restriction sites were added to the 5′ and 3′ ends of F30-2xdBroccoli so that it could be self-ligated to form tandem repeats. The strips of *Bst*EII-*Sph*I*-Spe*I restriction sites were also added to both ends of the repeats (Fig. 2B). The largest cassette contained 3 sets of F30-2xdBroccoli (12 copies) and was inserted into each of the 3 restriction sites. The correct insertion of 12xBroccoli at the 3′UTR site could not be recovered so the maximal number of Broccoli copies was 28 in TEnsP3–12x-ds12x-3′UTR4xBroccoli (TEds28Br) (Fig. 3).

### Signal intensity was brighter than Spinach2 and correlated with Broccoli copies in viral RNA

**Figure 4:**
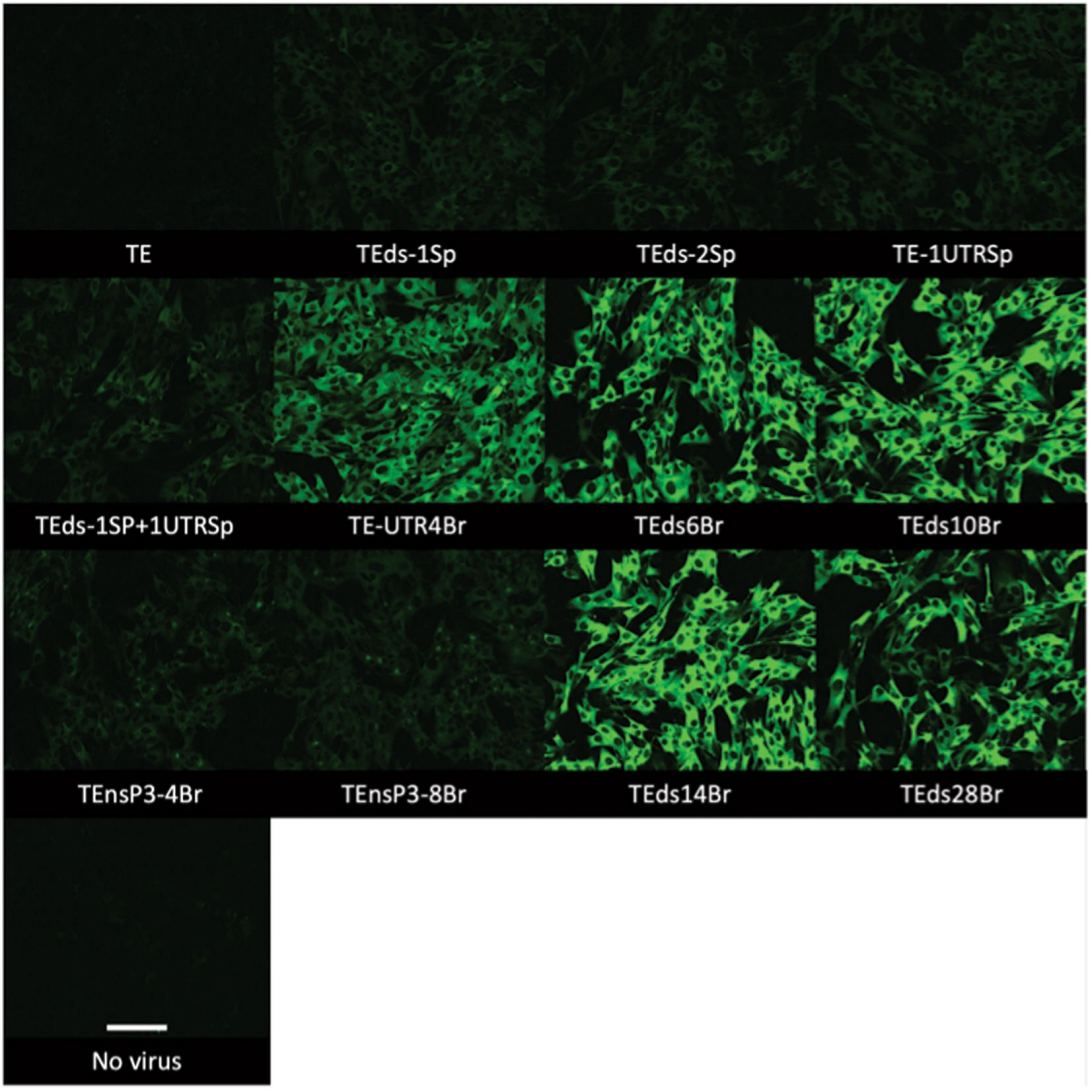
Comparison of signal intensities of all Spinach2 and Broccoli aptamer-tagged SINVs. The live cell images (objective lens 20×) compare signal intensities of all Spinach2 (Sp) and Broccoli (Br) recombinant viruses after adding DFHBI-1T to the infected BHK cells (MOI = 5, 6 h after infection). Scale bar = 100 μm.

To assess the effect of Broccoli copy number on signal intensity and compare the signal to that obtained with Spinach2, BHK cells infected with Spinach2-tagged and Broccoli-tagged SINVs were compared (Fig. 4). Viral RNA tagged with F30-2xdBroccoli is brighter than tRNA-Spinach2 although all viruses except TEds-2Sp replicate well in BHK cells^17^. For the Broccoli-tagged viruses, the signal intensity increased as numbers of Broccoli insertions increased from 4 to 14 copies. However, TEds28Br, which had tandem repeats of F30-2xdBroccoli, did not show a brighter signal than TEds14Br. Because the TEnsP3-4Br and TEnsP3-8Br had Broccoli only in genomic RNA, infected cells were not as bright as those infected with SINV that had Broccoli in the greatly amplified subgenomic RNA. These data indicated that the ability to insert more copies of the Broccoli aptamer compared to the Spinach aptamer into viral RNA improved visualization of RNA in infected cells.

### Broccoli signal survived fixation and co-localized with SINV RNA fluorescence *in situ* hybridization (FISH)

**Figure 5:**
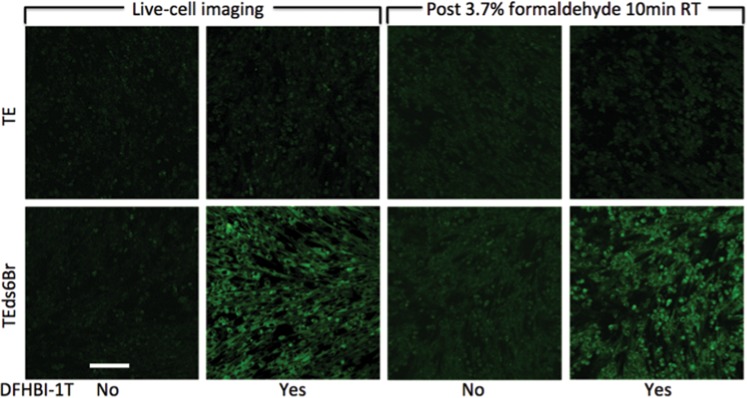
The effect of formaldehyde fixation on the Broccoli signal. The images (objective lens 20×) show BHK cells infected (MOI = 5) with TE (upper) or TEds6Br (lower) at 16 h after infection. Panels on the left are live cell images with and without addition of DFHBI-1T. Shortly after imaging, the same cells were fixed with 3.7% formaldehyde and re-imaged (panels on the right). Scale bar = 100 μm.

To determine whether Broccoli-tagged RNAs were also suitable for imaging fixed cells, BHK cells infected with TE-ds6Br were fixed with 3.7% formaldehyde for 10 min (Fig. 5). Unlike Spinach2, fixation did not obliterate the Broccoli signal, but did decrease the signal intensity and increase autofluorescence. Thus, live cell imaging provided a better signal to noise ratio than fixed cell imaging, but fixed cell imaging was possible.

**Figure 6:**
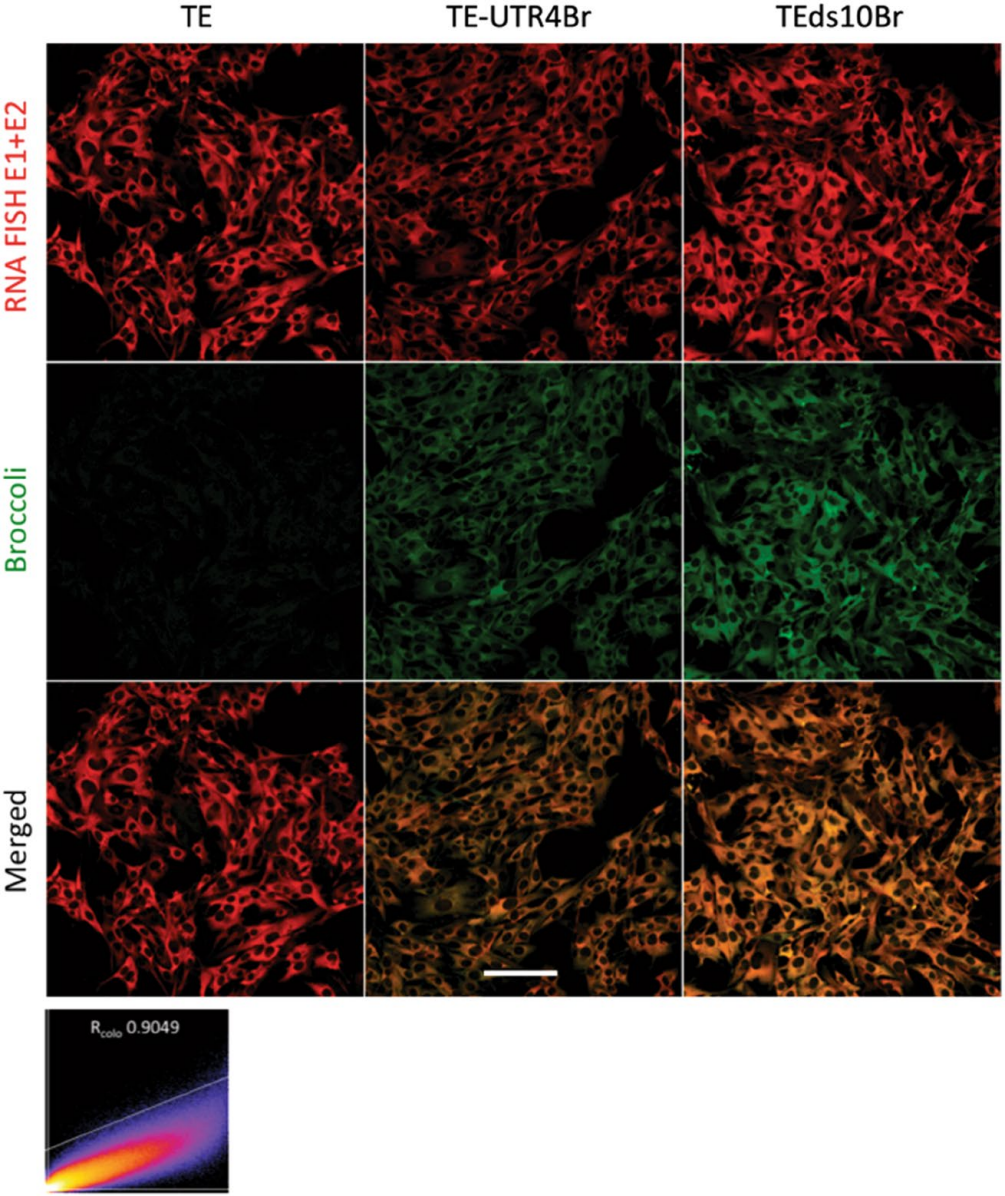
Colocalization between SINV RNA FISH and Broccoli. The images (objective lens 20×) show infected BHK cells (MOI = 5), fixed with 3.7% formaldehyde in PBS 6 h after infection. After adding DFHBI-1T, RNA FISH E1 + E2 (red) colocalizes well with Broccoli-DFHBI-1T (green) signal for both TEUTR4Br and TEds10Br. The scatter plot (X – red, Y – green) shows strong colocalization (R_colocalization_ = 0.9049). Scale bar = 100 μm.

**Figure 7:**
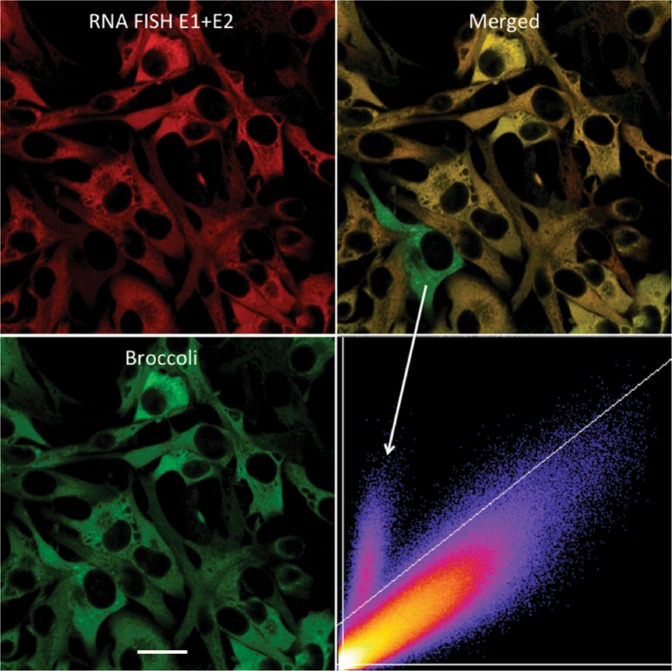
Rare discrepancy between RNA FISH and Broccoli signals. The images (objective lens 63×) show TEds10Br-infected BHK cells (MOI = 5), fixed with 3.7% formaldehyde in PBS at 6 h after infection. Although rare, some cells showed a discrepancy between FISH and Broccoli signals (white arrow), resulting in a separated cluster in the scatter plot (X – red, Y – green). Scale bar = 20 μm.

To compare imaging of SINV RNA by fluorescent *in situ* hybridization (FISH) with Broccoli-tagged SINV RNA, BHK cells were infected with TEUTR4Br and TEds10Br followed by FISH probes detecting sequences in E1 and E2 (Fig. 6). Post fixation imaging of infected BHK cells showed almost identical patterns between RNA FISH and Broccoli signals (R_colocalization_ = 0.9049) with only rare discrepancies for TEds10Br (Fig. 7) perhaps due to Broccoli insertion behind a second subgenomic promoter. RNA FISH tended to be brighter than aptamer-tagged RNA.

### Replication of SINV with a high Broccoli copy number was attenuated in differentiated neurons and mouse brain

**Figure 8:**
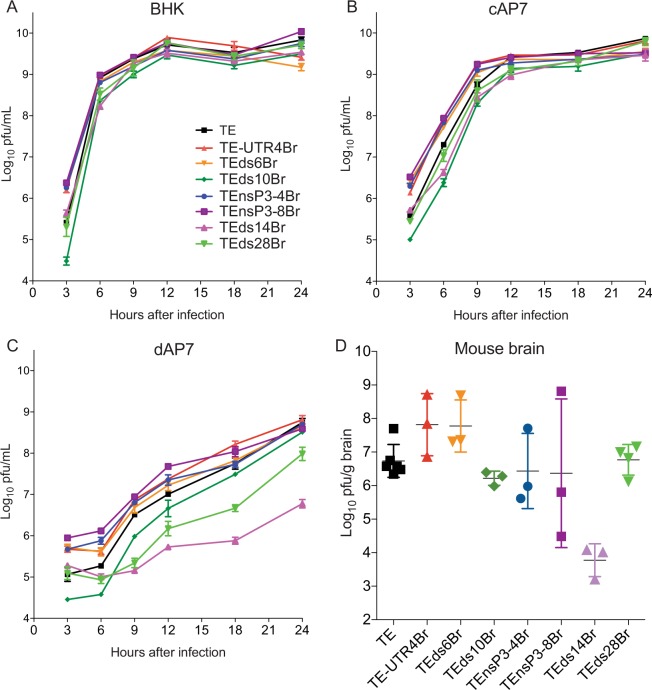
Replication of SINV-Broccoli in BHK and AP7 cells and in mouse brain. The graphs show the amount of infectious virus in supernatant fluids from BHK (**A**), cAP7 (**B**), or dAP7 (**C**) cells infected (MOI = 5) with SINV-Broccoli recombinant viruses (3 replicates per virus per time point). *P < 0.05; **P < 0.01; ***P < 0.001 (2-way ANOVA). Limit of detection was 50 pfu/ml (log_10_ 1.7). (**D**) Amount of infectious virus in brain homogenates from 4–6 week-old C57BL/6 mice 3 days after intracerebral infection with 1000 pfu SINV-Broccoli recombinant viruses (3–6 mice per virus).

To determine whether SINV replication was compromised by the Broccoli insertions, virus growth in BHK cells, undifferentiated cAP7 and differentiated dAP7 neural cells and in mouse brain was compared to TE (Fig. 8). All Broccoli-tagged SINVs replicated well in BHK cells (Fig. 8A). Viruses with Broccoli insertions in nsP3 replicated as well or better than untagged TE in cAP7 (Fig. 8B) and dAP7 (Fig. 8C) neuronal cells, but inconsistently in mouse brains with variable amounts of infectious virus detected 3 days after infection (Fig. 8D). TE3UTR4Br and TEds6Br replicated as well as untagged TE in neuronal cells (Fig. 8B,C) and in the brains of mice (Fig. 8D). On the other hand, TEds10Br, TEds28Br, and TEds14Br replicated less well in dAP7 cells (Fig. 8C) and mouse brains (Fig. 8D), but only marginally less well in cAP7 cells (Fig. 8B). Therefore, insertion site and number of insertions affected SINV replication in less permissive neural cells *in vitro* and *in vivo* more than in very permissive cells.

### Broccoli-tagged SINV can be visualized in differentiated neurons

**Figure 9:**
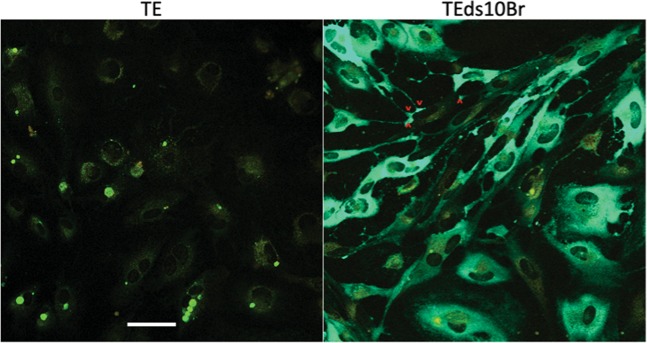
Accumulation of viral RNA in the foot processes of differentiated neurons. Live cell imaging (objective lens 20×) in lambda mode (spectral imaging) of TE (control) and TEds10Br-infected dAP7 cells (MOI = 20, 24 h after infection) plus DFHBI-1T. Viral RNA (green – Broccoli-DFHBI-1T) accumulated at filopodial tips (red arrow heads) in contact with an uninfected cell (yellow autofluorescence). Scale bar = 100 μm.

**Figure 10:**
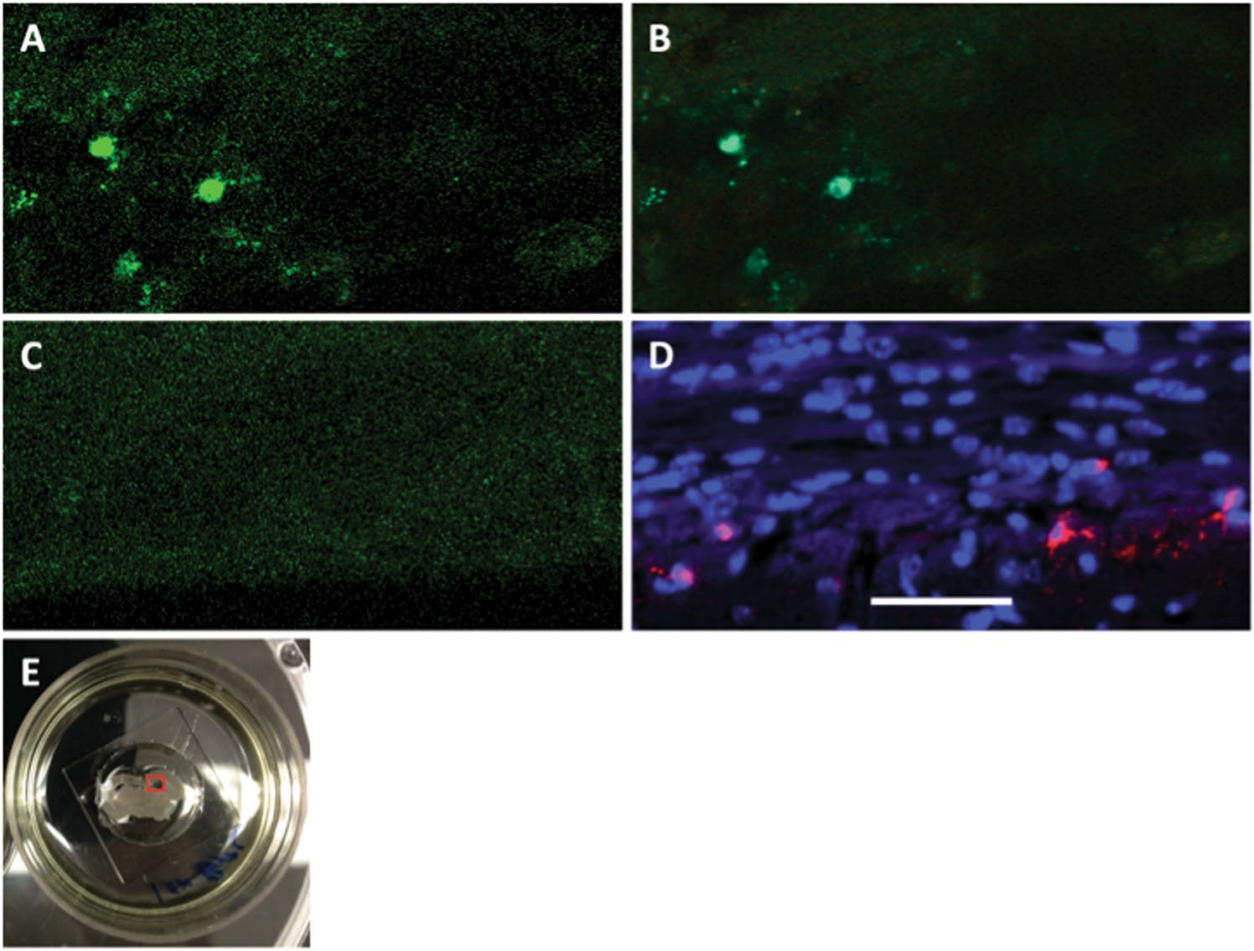
Live cell imaging of infected cells in mouse brain slices incubated with DFHBI-1T. (**A**) Channel mode and (**B**) lambda mode imaging of fresh TEds10Br-infected brain or (**C**) TE-infected brain. Broccoli-DFHBI-1T signal (green) in cells of the corpus callosum. (**D**) RNA E1 FISH imaging (red) of infected neurons in formalin-fixed brain tissue counterstained with DAPI. (**E**) Image of the 250 μm slice (McIlwain tissue chopper) of TEds10Br-infected mouse brain (3 days after infection) in a 35-mm glass-bottom dish in imaging medium containing DFHBI-1T with area of imaging for A, B indicated in the red square. Scale bar = 50 μm.

Spinach2-tagged SINV gave a very dim signal in differentiated neurons and spectral imaging (lambda mode) could differentiate the signal from autofluorescence in only a few of the brightest cells^17^. To assess the ability to visualize Broccoli-tagged SINV in differentiated neurons, we analyzed dAP7 cells infected with TEds10Br (Fig. 9). TEds10Br-infected dAP7 cells were bright enough for RNA to be visualized in most infected cells although lambda mode was still required. The Broccoli signal could also be observed in a brain slice obtained from a mouse 3 days after intracerebral infection with TEds10Br (Fig. 10A,B).

### Distribution of SINV genomic and subgenomic viral RNAs in BHK cells

**Figure 11:**
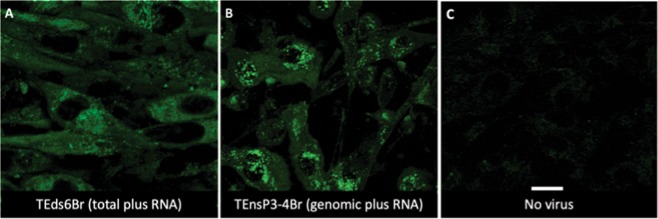
Distribution of SINV genomic and subgenomic RNA in BHK cells. (**A**) Live cell imaging (objective lens 63×) of TEds6Br-infected BHK cells (MOI = 5, 16 h after infection) shows more diffuse distribution of viral RNA (green – Broccoli-DFHBI-1T) when both genomic and subgenomic viral RNAs are labeled with Broccoli. (**B**) Cells infected with TEnsP3-4Br with labeled genomic RNA show focal accumulations of RNA in the perinuclear area. (**C**) Control image of uninfected cells incubated with DFHBI-1T. Scale bar = 20 μm.

**Figure 12:**
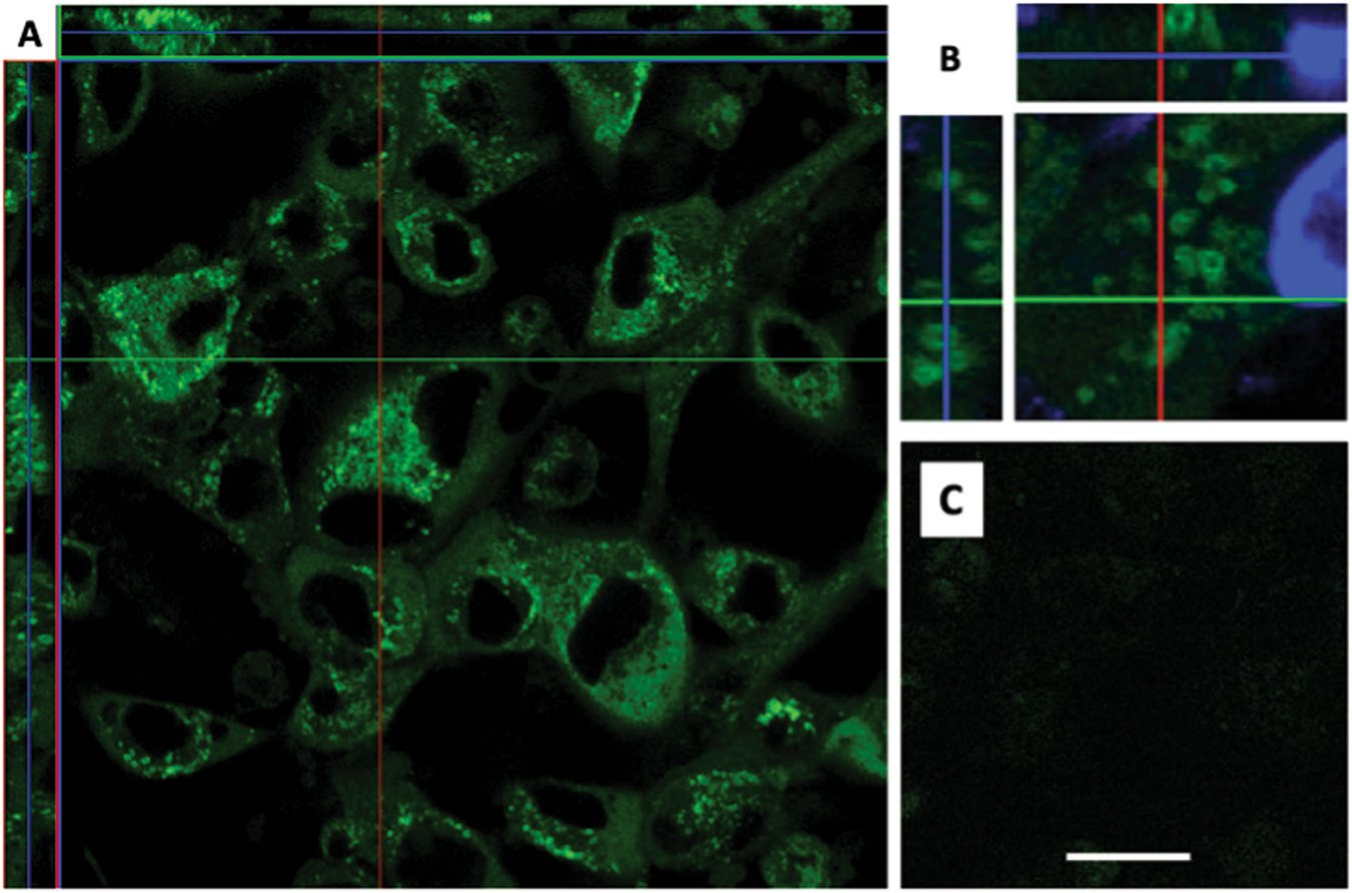
Vesicle-like arrangement of viral RNA. Live cell imaging of TEds14Br-infected BHK cells (MOI = 5, 20 h after infection) shows perinuclear vesicle-like arrangement of viral RNA (green – Broccoli-DFHBI-1T) in orthogonal view (**A,B** – zoom in). (**C**) TE-infected cells incubated with DFHBI-1T. Scale bar = 20 μm (**A**,**C**); 5 μm (**B**).

**Figure 13:**
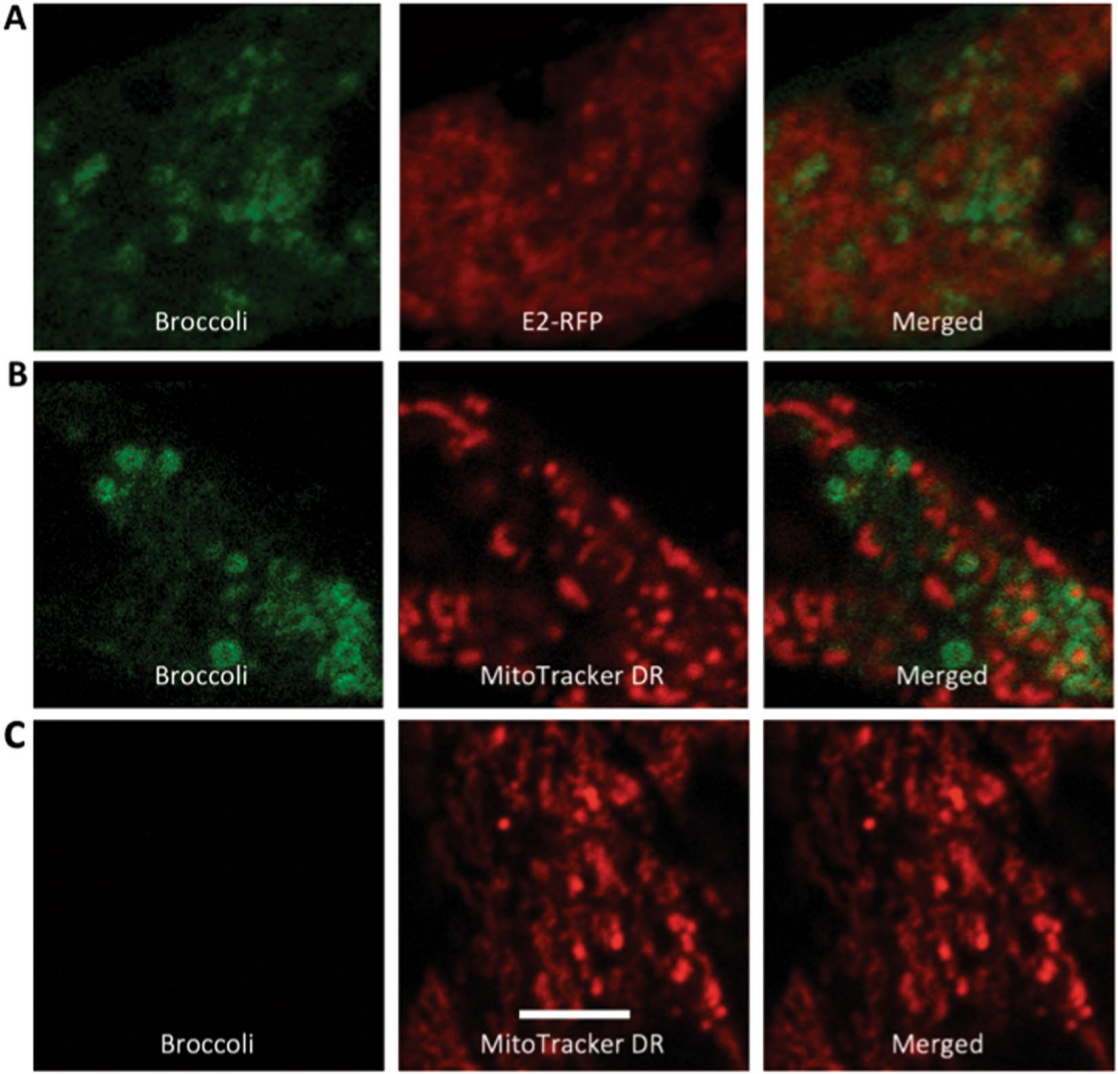
Viral RNA-associated structures. Live cell imaging (objective lens 63×) of TEds14Br-infected BHK cells (MOI = 5, 20 h) in cells co-infected with SINV expressing mCherry-tagged E2 protein^26^ (**A**) and in cells pre-labeled with Mitotracker DR 1 h before infection (**B**). (**C**) Uninfected cells labeled with MitoTracker DR. Scale bar = 5 μm.

**Figure 14:**
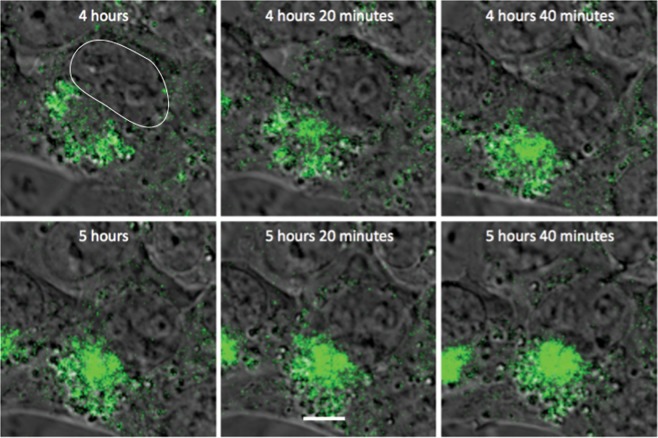
Accumulation of SINV genomic RNA at the perinuclear halo. Time-lapse live cell imaging (objective lens 63×) of TEnsP3-8Br-infected BHK cells (MOI = 20, 4–6 h after infection) shows genomic RNA-associated vesicles/vacuoles (green) surrounding the perinuclear halo (most likely the Golgi apparatus). Nucleus outlined in the initial image. Scale bar = 5 μm.

To localize genomic and subgenomic viral RNAs, BHK cells infected with TEnsp3–4Br (genomic) and TEds6Br (genomic and subgenomic) were imaged 16–20 h after infection (Fig. 11). Broccoli-tagged subgenomic RNA remained diffusely distributed in the cytoplasm (Fig. 11A), while at later times after infection Broccoli-tagged genomic RNA accumulated near the nucleus and assumed a circular shape with an inside vacuole (Figs. 11B and 12). To determine the relationship of this RNA to viral structural proteins required for virion assembly, cells were co-infected with TEds14Br and SINV expressing mCherry-tagged E2^26^ (Fig. 13A). Genomic RNA was present in close association with virion surface glycoprotein E2. To also determine the relationship of genomic RNA to mitochondria, cells infected with TEds14Br were stained with mitotracker dye to identify mitochondria (Fig. 13B). Co-staining showed genomic RNA associated with mitochondria. Time-lapse imaging of merged phase contrast and Broccoli showed many vesicles surrounded by SINV genomic RNA gathered near a perinuclear halo most likely to be the Golgi apparatus (Fig. 14).

## Discussion

Live cell imaging of viral RNA provides many advantages over fixed cell imaging at single time points, but has been difficult to accomplish. Previous studies of SINV RNA labeled with the Spinach2 aptamer were limited by the availability of insertion sites and brightness of the signal^17^. The current study has shown that labeling SINV RNA with multiple copies of the F30-2xdBroccoli aptamer overcomes many of the obstacles previously encountered with the Spinach2 aptamer. F30-2xdBroccoli is smaller and much brighter than tRNA-Spinach2 and, unlike the tRNA scaffold, the F30 scaffold is not a target for intracellular cleavage and does not contain stop codons^21^.

Lack of stop codons in the cassette allowed insertion into the genomic RNA region that encodes the nonstructural polyprotein. Viruses with 4–8 Broccolis in the C-terminal coding region of nsP3 replicated well and allowed visualization of genomic RNA. Like Spinach2^17^, insertion of 4 Broccolis into the 3′UTR did not compromise virus replication. At this site, as well as after a second subgenomic promoter, the RNA that is visualized is primarily subgenomic RNA because of its greater abundance. However, also like Spinach2, insertion of too many Broccoli copies (14 or more) impaired virus replication in differentiated cells and *in vivo*.

The greater brightness of the Broccoli signal also improved the quality of the imaging. Spinach2 can only be used for live cell imaging of SINV sgRNA in highly permissive undifferentiated cells (~5 × 10^5^ molecules/undifferentiated cell^27^). Multiple copies of Broccoli inserted into the nsP3 coding region, permitted visualization of genomic RNA (~1.6 × 10^5^ molecules/undifferentiated cell^28^) and viral RNA after infection of less permissive differentiated neural cells (Table 1).

**Table 1:** Summary of the utility of aptamer-tagged SINVs.

SINV-aptamer-fluorophore	BHK/cAP7		dAP7		Brain	
	Live	Fixed	Live	Fixed	Live	Fixed
Subgenomic-Spinach2-DFHBI	Poor	No	No	No	No	No
Subgenomic-Spinach2-DFHBI-1T	Fair	No	Poor	No	No	No
Subgenomic-Broccoli-DFHBI-1T	Good	Good	Fair	No	Poor	No
Genomic-Broccoli-DFHBI-1T	Fair	Poor	Poor	No	No	No
Dual-Broccoli-DFHBI-1T	Good	Good	Poor	No	No	No

In this study, we demonstrated at least two types of genomic RNA-containing vesicle-like arrangements in live cells. The one found earlier is most likely the endosome-derived cytopathic vacuole (CPV) type I, which is formed at the plasma membrane and contains the viral replication complex in spherules^29^. The genomic RNA near this vacuole has likely been exported from the spherule-associated replication complex that synthesizes a full-length minus-strand template for amplification of genomic viral RNA after initiation of infection^30,31^. Another vesicle type, CPV type II, was found in the perinuclear area in association with the trans Golgi network and endoplasmic reticulum. This vesicle near sites of sgRNA translation contains envelope proteins, is surrounded by viral nucleocapsids and likely the preparatory site for virion assembly^32^. Genomic RNA was also located around mitochondria that might be another type of cytopathic vacuole^33^ or reflect the interaction of viral RNA and protein with damaged mitochondria that accumulate in the perinuclear area prior to mitophagy^34^.

In summary, the production of virally encoded fluorescent RNA aptamers will facilitate new approaches for study of viral RNA dynamics *in vitro* and *in vivo*. The real-time measurement of the fluorescent signal intensity is faster, cheaper, easier and more informative than FISH or RT-qPCR. This technique is also suitable for high throughput anti-viral drug discovery that targets viral RNA replication.

## Materials and Methods

### Viruses

SINV Toto E2-mCherry was a gift from Richard Kuhn (Purdue University, West Lafayette, IN)^26^. SINV cDNA clones TE^22^ and TEds^23^ were the background for all aptamer-tagged recombinant viruses. After sequencing to confirm the correct insertions and orientations, each constructed plasmid was linearized with *Xho*I (New England Biolabs) and transcribed into mRNA by Sp6 mMessage mMachine (Ambion). Recombinant viruses were recovered by transfection of mRNA into BHK cells and collection of supernatant fluids after 24 h as previously described^17^. Titers of virus stocks were determined by plaque assay in BHK cells.

### Cell culture, differentiation and infection

BHK-21 cells were cultured at 37 °C 5% CO_2_ in DMEM-10% FBS. AP7 odora cells (gift from Dale Hunter, Tufts University, Boston), derived from rat olfactory neurons and immortalized with a temperature-sensitive SV40 T antigen^35^, were either passaged as cycling cells (cAP7) in DMEM-10% FBS at 33 °C 7% CO_2_, or were differentiated for 7 days into neurons at 39 °C 5% CO_2_ (dAP7) by adding 1 μg/ml insulin (Sigma), 100 μM ascorbic acid (Sigma), and 20 μM dopamine (Sigma) into the medium as previously described^36^. Cells were infected with the recombinant viruses in DMEM-1% FBS at multiplicities of infection (MOI) of 5 to 100. Replication was assessed in BHK, cAP7, and dAP7 cells after infection at an MOI of 5 by assaying supernatant fluids collected in triplicate by plaque assay in BHK cells.

### Live cell imaging

Cells were grown in 35 mm dishes, glass bottom 24-well plates (MatTek), or 8-well chamber cover glasses (Lab-Tek). Infected cells were imaged 30 min after replacing the culture media with phenol-red-free imaging medium (Fluorobrite, Gibco) containing 20 μM DFHBI-1T (Lucerna), 25 mM HEPES (Gibco) at 37 °C, 1% FBS, 1% PS and 2 mM glutamine. Autofluorescence was assessed relative to SINV TE (no aptamer)-infected cells and was minimized by adjusting the laser power and gain in each experiment. The laser scanning confocal microscope (Zeiss LSM780FCS, Axio Observer Z1, Zen Software) with A 488 nm laser excitation was used to activate the Broccoli-DFHBI-1T complex. In dAP7 cells, the true signal was differentiated from autofluorescence with the lambda scan that captured an emission spectrum in each pixel. The Broccoli-DFHBI-1T signal had an emission peak at 507 nm and appeared green in lambda mode, while the autofluorescence signal had no peak, extended from green to far red in its spectrum, and appeared yellow. In time-lapse experiments, temperature and CO_2_ were kept at the same level as the culture condition for each cell type with a heated chamber at 24% humidity. ImageJ Software was used for image analysis.

### Fluorescence *in situ* hybridization (FISH) of SINV RNA

BHK cells in a 96-well glass-bottom plate (Cellvis) infected with TEUTR4Br or TEds10Br were fixed with 3.7% formaldehyde in RNase-free PBS at room temperature for 10 min. After washing twice with RNase-free PBS, 5 min each, cells were permeabilized with 70% ethanol at 4 °C for 1 h. Ethanol was replaced with washing buffer (10% deionized formamide in 2x saline-sodium citrate [SSC]) for 5 min. Cells in each well were then incubated with two custom Stellaris RNA FISH probe sets each containing 48 short Quasar 570-labeled non-overlapping oligonucleotides against SINV E1 and E2 genes (LGC Biosearch Technologies), 25 nM each, in hybridization buffer (working buffer plus 10% dextran) at 37 °C overnight. Each well was then washed twice at 37 °C, 30 min each, with washing buffer. After another 2 washes with RNase-free PBS, 5 min each, imaging medium containing DFHBI-1T was added. The SINV RNA FISH (red) and Broccoli (green) signals were analyzed using the Co-localization Analysis plugin in ImageJ.

### Infection of mice

Three to six C57BL/6 mice, 4–6 weeks old, were infected intracerebrally with 1000 pfu/20 μL PBS of each recombinant virus. Three days after infection, brains were collected and the left anterior quadrant was homogenized with 1 ml PBS in Lysing Matrix A tubes in a FastPrep-24 machine (MP Biomedicals) at 6 m/sec speed for 40 sec to make 10% brain homogenates for plaque assays on BHK cells. All mouse studies were performed according to protocols approved by the Johns Hopkins University Animal Care and Use Committee.
